# Supplementary material for: Advancing ensemble learning techniques for residential building electricity consumption forecasting: Insight from explainable artificial intelligence
Source: PLoS One. 2024 Nov 14;19(11):e0307654. doi: 10.1371/journal.pone.0307654 (PMC11563398; doi:10.1371/journal.pone.0307654)
Supplement: Attachment — Submitted filename: Response to Review Comments_plos one.docx [file pone.0307654.s001.docx]

**Responses to Reviewers’ Comments**

We would like to express our sincere appreciation to the four anonymous reviewers for their valuable comments. Their comments and suggestions really helped us to improve the quality of our paper. We have done our best to address the issues raised by the reviewers and believe that we have successfully completed the additional work required. The newly added or significantly modified parts of the revised text are highlighted in red in the manuscript. Detailed responses to the reviewers' comments and the corresponding changes to the manuscript are provided below.

# Reviewer 1

**Comment 1:**

In my view, your literature review requires reconsideration and reorganization. Currently, you've listed recent papers employing deep learning algorithms while presenting the black box nature of these algorithms and the SHAP technique using decision tree-based algorithms as a novelty to address this. I concur that deep learning methods pose challenges for explanation, but recent advancements utilizing these algorithms may outperform decision tree-based algorithms in terms of accuracy. Hence, it's essential to distinguish between algorithmic performance superiority and explainability. Other studies might not explain their models but could achieve more reliable and accurate predictions. Thus, a discussion on the performance debate of these methods is warranted. Following this, you can emphasize the practicality and potential superiority of decision tree-based algorithms (if supported by literature), then discuss recent papers employing SHAP with tree-based models. I've come across a couple of works on this when searching Scopus. Subsequently, highlight the additions you've made to the existing literature. Your paper may not be the first to explore tree-based models with SHAP for forecasting electricity consumption. Hence, additional efforts are needed to establish your novelty and contributions.

**Response to comment:**

We would like to express our sincerest gratitude for your insightful and constructive feedback, which has been instrumental in refining our manuscript. Your critical observations prompted a thorough reassessment and reorganization of our literature review, compelling us to clearly distinguish between the performance and explainability of decision tree-based ensemble learning versus deep learning.

In response to your suggestions, we expanded our analysis to include a broader spectrum of XAI techniques beyond SHAP, enhancing our comparative study and providing a richer, more nuanced discussion of how these models handle complex datasets. This enhanced analysis has not only clarified the distinctive advantages and limitations of each modeling approach but has also identified the conditions under which each model excels, supported by rigorous empirical data.

Furthermore, your feedback prompted us to examine the practical implications of these technologies in greater depth. In particular, we have focused on the potential advantages and superiority of decision tree-based ensemble learning algorithms in specific scenarios, as supported by recent literature. This approach has significantly enhanced the value and originality of our research, differentiating it from existing works and showcasing its unique contributions to the field of energy management.

We have addressed each of your points in detail, ensuring that our manuscript meets and exceeds the standards of rigorous academic inquiry. The revisions made in response to your comments have significantly enhanced the paper’s clarity, depth, and scholarly value. We are confident that these comprehensive enhancements will meet the expectations of the scholarly community and contribute to the advancement of AI in energy forecasting.

We would like to thank you again for your invaluable feedback, which has been instrumental in improving our work. We are confident that the revisions made have robustly addressed your concerns and enriched the manuscript, ensuring that it makes a significant contribution to the field.

**Comment 2:**

In the abstract, the first, the second, and the last sentences convey similar messages. Instead, consider emphasizing the research gap and lessons learned.

**Response to comment:**

Thank you for your valuable feedback on our manuscript. We have carefully considered your advice to emphasize the research gap and lessons learned in the abstract, rather than repeating similar messages. In response, we have revised the abstract to more effectively highlight the distinctive contributions and findings of our study.

- We have shifted the focus to the critical role of accurate electricity consumption forecasting in residential buildings for enhancing energy efficiency and cost management, which is fundamental to sustainable energy practices.
- We have demonstrated the effectiveness of decision tree-based ensemble learning techniques, enhanced with explainable artificial intelligence (XAI), in processing complex datasets with high accuracy and providing clarity and interpretability.
- Furthermore, in alignment with your suggestion for practical applicability, we have made the complete study and source code publicly available on our GitHub repository, facilitating transparency, replication, and real-world application of our research findings.

We are confident that these revisions and actions directly address your concerns and greatly enhance the manuscript's contribution to the field. We trust that these changes meet your expectations and that you will be pleased with the revised manuscript.

**Comment 3:**

Table 2 needs relevant citations to substantiate the claims made.

**Response to comment:**

Thank you for your valuable feedback regarding Table 2. We have incorporated relevant citations to substantiate the claims made, referencing the following papers:

- **[Random Forest]** Fox EW, Hill RA, Leibowitz SG, Olsen AR, Thornbrugh DJ, Weber MH. Assessing the accuracy and stability of variable selection methods for random forest modeling in ecology. Environ Monit Assess. 2017; 189: 1–20.
- **[Random Forest]** Zhang Y, Liu J, Shen W. A review of ensemble learning algorithms used in remote sensing applications. Appl Sci. 2022; 12: 8654.
- **[Gradient Boosting Machine]** Srivastava S, Lopez BI, Kumar H, Jang M, Chai HH, Park W, et al. Prediction of Hanwoo cattle phenotypes from genotypes using machine learning methods. Animals. 2021; 11: 2066.
- **[Gradient Boosting Machine; Extreme Gradient Boosting; Light Gradient Boosting]** Mienye ID, Sun Y. A survey of ensemble learning: Concepts, algorithms, applications, and prospects. IEEE Access. 2022; 10: 99129–99149.
- **[Extreme Gradient Boosting; Light Gradient Boosting]** González S, García S, Del Ser J, Rokach L, Herrera F. A practical tutorial on bagging and boosting based ensembles for machine learning: Algorithms, software tools, performance study, practical perspectives and opportunities. Inf Fusion. 2020; 64: 205–237.
- **[Categorical Boosting]** Hancock JT, Khoshgoftaar TM. CatBoost for big data: an interdisciplinary review. J Big Data. 2020; 7: 94.

Furthermore, in light of the revisions to our manuscript, this table is now presented as Table 3. We would like to express our gratitude for your invaluable suggestions, which have helped us to improve the rigor and credibility of our work. Thank you for your guidance.

**Comment 4:**

I suggest moving the definitions of performance metrics to the methods section, as these definitions do not constitute results.

**Response to comment:**

Thank you for your valuable suggestion regarding the placement of the performance metric definitions.

We have moved these definitions to the "Forecasting model development" section in accordance with your advice. To further enhance the clarity and logical structure of our manuscript, we have created a new subsection titled "Performance metrics and hyperparameter optimization" under this section.

In this new subsection, we have included both the definitions of the performance metrics and the table for hyperparameter settings. We believe that this adjustment addresses your comment and improves the overall readability and coherence of our paper.

We appreciate your insightful comments and the opportunity to improve our work. Thank you once again for your thorough review and constructive feedback.

**Comment 5:**

Hyperparameter tuning necessitates a table displaying your grid search intervals and tuning parameters. This is essential for a machine learning-based study.

**Response to comment:**

Thank you for your insightful suggestion to include a table detailing our grid search intervals and tuned parameters for hyperparameter tuning. As recommended, we have added Table 4 to our manuscript, which follows the description of our hyperparameter optimization process.

This table provides a comprehensive overview of the grid search intervals and final tuned parameters used in our study, ensuring transparency and reproducibility of decision tree-based ensemble learning methods.

**Table 4.** List of hyperparameters for decision tree-based ensemble learning method.

| **Methodologies** | **References** | **Hyperparameters** |
| --- | --- | --- |
| Random forest | [52] | - Trees count: 128 - Features per split: auto, sqrt, log2 |
| Gradient boosting machine | [41] | - Iteration count: 100, 250, 500 - Learning rate: 0.01, 0.05, 0.1 - Depth: 5, 10 - Loss type: quantile, Huber |
| Extreme gradient boosting | [41] | - Iterations: 250, 500, 1000 - Learning rate: 0.01, 0.05, 0.1 - Depth: 6, 8, 10 - Subsampling rate: 0.5, 0.75, 1.0 - Feature sample by tree/level/node: 0.5, 0.75, 1.0 - Booster type: gbdt, dart |
| Light gradient boosting machine | [41] | - Iterations: 1000, 1500 - Learning rate: 0.01, 0.05, 0.1 - Leaves: 64 - Subsample: 0.5 - Feature sample by tree: 1.0 - Booster type: gbdt, dart |
| Categorical boosting | [53] | - Learning rate: 0.03, 0.1 - Max tree depth: 4, 6, 10 - L2 regularization levels: 1, 3, 5, 7, 9 |

We believe this addition significantly enhances the clarity and depth of our methodology section, allowing readers to fully understand the rigor of our optimization process. We appreciate your help in making our study more robust and accessible.

**Comment 6:**

Please reconsider the captions for tables 3, 4, and 5. They are identical. Rewrite the captions to highlight the distinct content in each table, possibly utilizing the footnotes provided.

**Response to comment:**

Thank you for your suggestion to reconsider the captions for Tables 3, 4, and 5. We have revised the captions to highlight the distinct content of each table, utilizing the provided footnotes. Additionally, due to revisions in our manuscript, these tables are now presented as Tables 8, 9, and 10. Your feedback has been instrumental in enhancing the clarity and distinctiveness of our table captions.

In accordance with your guidance, we have updated the captions for Tables 8, 9, and 10 to more accurately reflect the distinct content in each table. Please find the updated captions below:

- Table 8: Comparative analysis of decision tree–based ensemble learning models trained with external factors on the Appliances Energy Prediction dataset.
- Table 9: Comparative analysis of decision tree–based ensemble learning models trained with internal factors on the Appliances Energy Prediction dataset.
- Table 10: Comparative analysis of decision tree–based ensemble learning models trained with external and internal factors on the Appliances Energy Prediction dataset.

We believe these changes enhance the clarity and specificity of our tables, thereby providing a clearer understanding of the comparative analyses presented. We would like to express our gratitude once again for your thorough review and constructive suggestions. Your input has been instrumental in enhancing the quality of our manuscript.

**Comment 7:**

The discussion section requires significant refinement. As a scholar who has employed the SHAP technique in nearly a dozen papers, I believe that key insights and lessons derived from these plots should be comparatively discussed with similar literature. The feature importance and contributions of input variables to the learning phase (whether positive or negative) as depicted in SHAP plots should be compared with recent studies. How do your findings corroborate or contradict existing research?

**Response to comment:**

Thank you for your valuable feedback regarding the discussion section. We have made significant revisions to this section to incorporate a comparative analysis with existing literature, focusing on the insights and lessons derived from SHAP plots.

We have included detailed comparisons of feature importance and contributions of input variables, contrasting our findings with recent studies to highlight how they corroborate or differ from existing research. Furthermore, we have discussed why our approach using SHAP is superior to other techniques such as LIME and PDP in terms of identifying and combining meaningful variables, as well as verifying the accuracy of generated models.

Specific improvements include:

- Comparative Analysis: We compared the variable importance from our SHAP analysis with results from similar studies, emphasizing both corroborations and contradictions.
- Methodological Superiority: We highlighted the advantages of SHAP over LIME and PDP, demonstrating its effectiveness in revealing complex interactions within the data.
- Practical Application: We provided a comprehensive overview of how SHAP was used to enhance model integrity and verify accuracy.

These enhancements ensure a more balanced and insightful discussion, aligning with your expertise and suggestions. We believe these changes significantly improve the rigor and depth of our manuscript. We would like to express our gratitude once more for your invaluable guidance, which has been instrumental in enhancing the quality of our work.

**Comment 8:**

Furthermore, consider whether Figures 11 and 12 truly belong in the discussion section or if they should be relocated to the results section.

**Response to comment:**

Thank you for your insightful suggestion regarding the placement of Figs 11 and 12. After careful consideration, we have relocated these figures to the "SHAP analysis of the optimal decision tree-based ensemble model" subsection within the "Experimental results and discussion" section. Additionally, due to revisions in our manuscript, these figures are now presented as Figs 12 and 13. We believe that this placement is more appropriate and aligns better with the content, providing a clearer and more logical structure to our manuscript.

By relocating these figures, we believe we have enhanced the clarity of our results and ensured that the discussion is more focused and coherent. We are confident that this adjustment improves the overall readability and organization of our paper.

**Comment 9:**

Your discussion section should include a segment dedicated to the algorithmic debate. How did you interpret the performance of RF, LightGBM, CatBoost, GBM, and XGBoost? Do you believe they offer superior performance compared to deep neural networks? Additionally, what are your thoughts on the practicality of employing the TreeSHAP library with decision tree-based models, and do you have any plans for future studies to enhance the significance of your work? To support your approach, could you provide other examples of papers utilizing these algorithms with SHAP? These examples need not be limited to your field; you can search for the 'algorithm name-SHAP' combination on search engines to identify similar methodologies across various disciplines. In my view, it's crucial to broaden the scope of your paper to encompass other domains, as each dataset may differ while employing the same machine-learning framework. A debate of this nature would undoubtedly yield valuable insights for an interdisciplinary journal.

**Response to comment:**

Thank you for your valuable feedback regarding the discussion section. We have significantly expanded this section to include a detailed comparative analysis of feature importance and contributions of input variables as depicted in SHAP plots, and contrasted our findings with recent studies.

The study examined the performance of ensemble models, including RF, GBM, LightGBM, and CatBoost, demonstrating their superiority over deep learning models in structured data scenarios. This aligns with the conclusions of Bellahsen and Dagdougui [17] and Zhang et al. [18]. CatBoost effectively utilized timestamp information, significantly reducing CVRMSE. The models demonstrated resilience to overfitting and efficient handling of noisy data, making them suitable for energy consumption forecasting. Strategies included applying ensemble learning models to various residential data environments, comparing multiple ensemble learning models with deep learning alternatives, and using diverse XAI techniques beyond SHAP.

We evaluated the performance of RF, LightGBM, CatBoost, GBM, and XGBoost, finding that these ensemble models often outperform deep neural networks, especially in handling variability and noise. The practical application of the TreeSHAP library provided deeper interpretive insights, revealing complex data interactions more effectively than LIME or PDP. For instance, our "SHAP analysis of the optimal decision tree-based ensemble model" section demonstrated how SHAP provided a more detailed analysis than traditional feature importance, enhancing interpretability and revealing intricate patterns (Fig. 4 and Fig. 7).

The TreeSHAP library proved to be a practical and insightful tool. Future studies will extend these models and SHAP to diverse domains and datasets to enhance generalizability. This approach has been successfully employed in other studies in finance and healthcare, where SHAP has been used with ensemble models to interpret complex data. Our findings corroborate studies by Moon et al. [32] and Lahouar and Slama [61] on the importance of historical consumption patterns. The nuances in the effects of variables like holidays and the temperature-humidity index also align with observations by Li et al. [62], which highlights the impact of dataset specifics and model configurations.

Our refined discussion highlights the efficacy of ensemble learning models in handling energy usage complexities. Focusing on tailored solutions and enhancing data-driven strategies can significantly improve predictive accuracy for both large-scale and small-scale scenarios. This analysis not only contributes to academic discourse but also provides strategic direction for future research, ensuring our models are effective, comprehensible, and applicable across various domains. We would like to thank you once again for your guidance and insightful suggestions, which have significantly enhanced our manuscript.

# Reviewer 2

The manuscript explores how explainable artificial intelligence can enhance decision tree–based ensemble learning methods for more effective short-term load forecasting in residential energy systems. The manuscript is written well and the analysis seems sufficient. The following comments must be addressed for possible acceptance of the manuscript.

**Comment 1:**

Why are these 80% training and 20% testing subsets considered? The usual ratio is 70:30.

**Response to comment:**

We are grateful for your astute observation regarding the training and testing subset ratios. We appreciate your suggestion and recognize that a 70:30 split is a commonly utilized approach in numerous studies. However, the initial ratio of 80:20 was selected based on empirical experience with regression problems and the objective of adequately capturing the correlations within the relatively short AEP dataset. Nevertheless, we recognize the value of adhering to established norms and have modified our methodology to align with these standards.

For the AEP dataset, we have revised the training and testing periods as follows:

- Training period: From January 11, 2016, 6:00 PM to April 16, 2016 (approximately 70% of the data)
- Testing period: From April 17, 2016, to May 27, 2016, 6:00 PM (approximately 30% of the data)

For the University Residential Complex dataset, we have adopted the 70:30 split as recommended:

- Training period: From September 1, 2015, to December 31, 2017 (28 months; 70%)
- Testing period: From January 1, 2018, to December 31, 2018 (12 months; 30%)

It is our contention that these adjustments are more in keeping with standard practices and will yield more robust and generalizable results. Your contributions have been of great assistance in guiding these improvements. We would like to express our gratitude once again for your comprehensive review and constructive suggestions.

**Comment 2:**

Different input variable configurations are not clearly explained. Mention them separately in the model development section.

**Response to comment:**

Thank you for your constructive feedback regarding our manuscript. Your specific comment on the clarity of the different input variable configurations in the model development section was invaluable. We recognize that the success of our study depends on the clarity and reproducibility of our methods, including how input variables are configured and utilized within our models.

In response to your feedback, we have significantly revised and expanded the “Forecasting model development” section of our manuscript. We have now clearly defined each input variable configuration used in our study. This context includes a detailed description of the variables selected for each model, the rationale behind these selections, and how these configurations impact the model's performance and forecasting accuracy.

Furthermore, we have included additional subsections, “Data gathering and preparation for model development,” that separately address the preprocessing of these variables and the specific techniques used to integrate them into our decision tree-based ensemble learning methods. This structured approach ensures that readers can easily understand the role each input variable plays in our analysis and how they can potentially replicate or build upon our work.

We hope that these revisions address your concerns and significantly contribute to the manuscript's overall quality and clarity. Thank you again for your valuable insights and guidance.

**Comment 3:**

In Fig. 11, insert a linear trendline in the scatter plots.

**Response to comment:**

Thank you for your astute observation regarding Fig 11. We have incorporated a linear trend line into the scatter plots in response to your valuable input. This modification enhances the clarity and interpretability of our data. Additionally, due to the revisions in our manuscript, this figure is now presented as Fig 12. Your expertise and guidance have been invaluable in making these improvements.

We appreciate your constructive feedback, which has undoubtedly enhanced the quality of our work. We hope that this amendment meets your expectations and further contributes to the comprehensiveness of our study. We would like to thank you once again for your thoughtful and beneficial input.

|  |  |  |
| --- | --- | --- |
| (a) | (b) | (c) |
|  |  |  |
| (d) | (e) | (f) |
| **Fig 12. Scatterplots and *R*^2^ comparison for each decision tree–ensemble learning model.** (a) random forest; (b) gradient boosting machine; (c) extreme gradient boosting; (d) light gradient boosting machine; (e) categorical boosting; (f) *R*^2^ comparison. | | |

**Comment 4:**

A theoretical overview of ensemble methods must be presented.

**Response to comment:**

We would like to express our gratitude for your valuable feedback, which has significantly enhanced the clarity and depth of our paper.

We fully concur with your perspective and have taken this opportunity to enhance our manuscript by creating a dedicated section entitled Comprehensive guide to ensemble learning methods." This new section provides a comprehensive theoretical overview of the ensemble methods used in our study.

Your constructive comments were instrumental in guiding these improvements. We would like to thank you again for your thorough review and the opportunity to improve our manuscript.

**Comment 5:**

Try incorporating Taylor Diagrams for comparative evaluation of models developed.

**Response to comment:**

Thank you for your insightful suggestion to incorporate Taylor Diagrams for a comparative evaluation of ensemble learning models. We have included Taylor diagrams in our revised manuscript to provide a comprehensive visual assessment of different ensemble learning techniques.

The Taylor diagram in Fig. 2 offers a detailed illustration of the performance of various ensemble models on the University Residential Complex dataset. It shows that most ensemble models achieved correlations above 0.9, indicating high accuracy and low RMSE values. This demonstrates their reliability and superior predictive accuracy.

**Fig 2.** Taylor diagram showing the performance of ensemble learning models on the University Residential Complex dataset.

Additionally, Fig. 3 presents the performance of ensemble learning models, with a focus on the RF model trained on the Appliances Energy Prediction dataset. Although the RF model achieved a moderate correlation of about 0.6, this comparison underscores the challenges and areas for improvement when transitioning from large to small-scale applications.

**Fig 3.** Taylor diagram showing the performance of ensemble learning models on the Appliances Energy Prediction dataset.

Your suggestion has significantly enhanced our comparative analysis, providing deeper insights into the models’ performance. We appreciate your guidance and support, which have been invaluable in improving our manuscript.

**Comment 6:**

Enhance the results and discussion part by comparative analysis with existing literature results.

**Response to comment:**

Thank you for your valuable feedback. We have enhanced the "Experimental results and discussion" section by incorporating a detailed comparative analysis with existing literature. Specifically, we compared the performance of our ensemble models with state-of-the-art deep learning and hybrid models, such as LSTM, GRU, TCN, and their combinations, in a new section titled "Comparison with state-of-the-art deep learning and hybrid models."

Our analysis revealed that while deep learning models demonstrated satisfactory performance, ensemble learning models, namely GBM and CatBoost, exhibited superior performance in handling diverse and noisy datasets with less extensive preprocessing (see Tables 13 and 14). For instance, the GBM model demonstrated the lowest MAPE, CVRMSE, and NMAE in structured data scenarios, underscoring its robustness and efficiency.

We are grateful for your insightful suggestions, which have significantly enhanced the depth and clarity of our manuscript. Your guidance has been invaluable in enhancing our comparative analysis and demonstrating the strengths of our models. We appreciate your support.

**Comment 7:**

Mention the limitations and future scope of work.

**Response to comment:**

Thank you for your valuable input regarding the limitations and future scope of our work. We have updated the "Conclusions" section to address these aspects.

Future research will expand the applicability of the proposed methodology to various building types, including commercial and industrial settings, to assess its adaptability and effectiveness across diverse environments. Furthermore, we intend to incorporate data on renewable energy sources and occupancy patterns to enhance the precision and relevance of the model. Furthermore, we will develop methods to address data heterogeneity and privacy concerns, and innovate an online dynamic learning-based energy forecasting model to enhance energy efficiency.

Our objective is to develop an advanced energy management and trading system based on robust demand and supply forecasting, incorporating external factors such as consumer behavior.

Thank you again for your comprehensive review and valuable suggestions. We are grateful for your valuable feedback, which has significantly enhanced the quality of our manuscript.

# Reviewer 3

**Comment 1:**

Areas for Improvement and Recommendations: Validation of Results: The study should include additional validation of the models with external datasets to ensure the robustness and generalizability of the findings. Consider employing other metrics for model evaluation to complement MAPE, CVRMSE, and NMAE for a more rounded assessment of model performance.

**Response to comment:**

Thank you for your valuable suggestions regarding the validation of our models and the enhancement of our evaluation metrics. Your recommendations have greatly contributed to the refinement of our research methodology and the robustness of our findings.

In response to your comments, we have extended the validation of our decision-tree-based ensemble learning models by incorporating external datasets. Specifically, we utilized data from a cluster of residential building dormitories at a private university in Seoul, South Korea. The incorporation of this dataset has enabled us to test the generalizability and robustness of our models in forecasting electricity consumption in residential buildings, enhancing the methodological transparency of our study.

Furthermore, to complement our initial model evaluation metrics (MAPE, CVRMSE, and NMAE), we have introduced the calculation of the harmonic mean (HM) of these metrics. The harmonic mean provides a balanced measure of model performance by effectively mitigating the impact of extreme outliers. This addition ensures a more comprehensive assessment, favoring models that perform consistently well across all considered metrics.

The following equations have been included in our empirical analysis to maintain transparency and provide clarity on our methodological approach:

- MAPE = 1 / n × ∑|(Yt – Ŷt)/Yt| × 100
- CVRMSE = (√(1 / n × ∑(Yt – Ŷt)^2))/Ÿ × 100
- NMAE = (1 / n × ∑|Yt – Ŷt|)/Ÿ × 100
- HM = (3 × MAPE × CVRMSE × NMAE) / (MAPE + CVRMSE + NMAE)

These metrics have been carefully chosen for their ability to provide a transparent and understandable assessment of accuracy for both technical and non-technical stakeholders. By incorporating these diverse metrics, we ensure that our evaluation process is robust, making it easier to identify models that are both reliable and accurate.

We believe these improvements address your concerns and significantly enhance the quality of our manuscript. Thank you again for your insightful feedback, which has been instrumental in strengthening our research.

**Comment 2:**

Comparison with State-of-the-Art: A comparison with the latest state-of-the-art methods in electricity consumption forecasting could strengthen the manuscript's contribution to the field. This includes deep learning and hybrid models not limited to decision tree–based methods.

**Response to comment:**

In response to your suggestion, we have included a new section in our manuscript titled "Comparison with State-of-the-Art Deep Learning and Hybrid Models." This section presents a comprehensive comparison of our forecasting models against advanced deep learning and hybrid architectures.

As illustrated in the attached overview of our experimental setup, we have employed a variety of deep learning models, including LSTM (long short-term memory), Bi-LSTM (Bidirectional LSTM), GRU (gated recurrent unit), Bi-GRU (Bidirectional GRU), 1D-CNN (one-dimensional convolutional neural network), and their combinations with TCN (temporal convolutional network), all enhanced with attention mechanisms. The models have been configured to predict the next time point in electricity consumption using a many-to-one approach over 24 time steps.

The results validate the superiority of our proposed decision tree-based ensemble models, which offer enhanced interpretability via SHAP analyses, computational efficiency without the need for GPUs, and strong performance on tabular datasets typical in energy forecasting. These factors make our models not only practical but also highly effective, combining ease of use with robust predictive capabilities, thereby setting a new benchmark in electricity consumption forecasting.

We believe that this addition substantially strengthens our manuscript by showcasing our methodology's alignment with cutting-edge research and its capability to push the boundaries of current forecasting technologies. Thank you once again for your insightful suggestions.

**Comment 3:**

Methodological Clarifications: The manuscript would benefit from a clearer explanation of the criteria for selecting the ensemble learning techniques analyzed. Including information on why certain methods were chosen over others would provide readers with a deeper understanding of the study's scope. Further elaboration on the data preprocessing steps and their impact on the results would enhance methodological transparency.

**Response to comment:**

Thank you for your constructive feedback on our manuscript. We appreciate your suggestion for a clearer explanation of the criteria for selecting specific ensemble learning techniques and the need for more detailed information on data preprocessing steps.

In response to your comments, we have revised the manuscript to include a more explicit explanation of the reasons behind the choice of each ensemble learning method analyzed. Specifically, we have expanded on how methods such as RF, GBM, XGBoost, LightGBM, and CatBoost were selected based on their unique capabilities to handle large datasets, reduce bias and variance, and their suitability for complex predictive modeling tasks. These clarifications are intended to provide readers with a deeper understanding of the study’s scope and the rationale behind the chosen methodologies.

Furthermore, we have expanded the section on data preprocessing to provide a detailed account of the steps taken before applying the ensemble learning techniques. This includes explanations on data cleaning, feature selection, normalization, and handling of missing values. We have described how each preprocessing step influences the model's performance and the overall results of the study. To further enhance methodological transparency, we have also included Table 4. This table provides a comprehensive overview of the hyperparameters used and their specific configurations, offering readers a clear view of our model tuning process.

We believe these revisions will significantly improve the clarity and transparency of our methodology, ensuring that the scientific community can fully understand and replicate our study. Thank you once again for your insightful suggestions, which have undoubtedly strengthened our manuscript.

**Comment 4:**

Discussion on Limitations: While the manuscript highlights the advantages of integrating XAI, a more detailed discussion on the limitations and challenges encountered during the study, including the handling of complex datasets and computational requirements, would provide a more balanced view.

**Response to comment:**

Thank you for your valuable feedback on the discussion section. We have carefully considered your suggestion to include a more detailed discussion on the limitations and challenges encountered during our study. In response, we have made the following revisions:

- We acknowledged the challenges posed by high variability and the presence of outliers in datasets such as the Appliances Energy Prediction dataset. We have expanded on the advanced preprocessing techniques used to construct realistic input variables and address data leakage, ensuring the reliability of our models.
- We discussed the computational demands associated with deep learning models and some ensemble methods, emphasizing the need for more efficient computational strategies, particularly for real-time applications. We emphasized the importance of extending the dataset's timeframe and including more diverse environmental and operational variables to capture broader trends and further test model adaptability under different conditions.
- We have outlined the significant challenges of integrating XAI, particularly SHAP, into complex models, including handling high-dimensional data and meeting computational demands. We have emphasized the necessity for advances in computational efficiency and algorithm optimization to make XAI more practical and accessible.

These additions aim to provide a balanced view of our study, acknowledging both its strengths and limitations. We believe these changes enhance the clarity and depth of our discussion, aligning with your insightful suggestions.

We would like to thank you once again for your constructive feedback, which has significantly improved our manuscript.

**Comment 5:**

Future Work: The conclusion section could be expanded to outline specific directions for future research, such as exploring the application of the proposed methodology to other types of buildings or integrating additional types of data (e.g., renewable energy sources, occupancy patterns).

**Response to comment:**

Thank you for your valuable feedback on our manuscript. We have carefully considered your recommendations to extend our research to additional building types and to integrate more diverse data sources. In response, we have expanded our discussion of future research directions. We plan to apply the proposed methodology to different building types, such as commercial buildings and industrial complexes, to explore its adaptability and effectiveness in different environmental and usage contexts. Furthermore, we will incorporate data on renewable energy sources and occupancy patterns into our models. This integration is expected to significantly enhance the precision and relevance of our predictions by more accurately reflecting dynamic energy generation and consumption scenarios.

We believe these enhancements will greatly enrich our research and contribute to a more comprehensive understanding of energy management systems. We appreciate your guidance in refining our focus and are excited about the potential impact of these expanded research directions. Thank you again for your valuable feedback.

# Reviewer 4

**Comment 1:**

The article has taken up well, but it can be written better by avoiding many short forms.

**Response to comment:**

Thank you for your valuable feedback and for your careful review of our manuscript. We appreciate your suggestion to avoid the use of many short forms in order to improve the clarity and readability of the article.

We have taken your advice to minimize the use of abbreviations and acronyms throughout the manuscript and have made a concerted effort to comply with the PLoS ONE author guidelines. In instances where abbreviations were necessary for conciseness and to avoid repetitive phrasing, we ensured that each abbreviation was clearly defined upon its first use.

This approach strikes a balance between readability and brevity, making the content more accessible to a broader audience, including those who may not be familiar with the specific terms used. Furthermore, we have reviewed and revised sections where abbreviations were previously overused, replacing them with full terms where possible.

We believe these revisions have significantly enhanced the manuscript's readability and overall quality. We hope that these efforts meet your expectations and contribute to a more accessible and well-written article.

**Comment 2:**

The authors have taken the harmonic mean of three matrices in the experimental results section. Is there any specific reason for using the harmonic mean?

**Response to comment:**

Thank you for your insightful feedback and for highlighting the use of the harmonic mean in our analysis. We appreciate the opportunity to clarify our methodology.

In our empirical analysis, we evaluated the effectiveness of the DT-EL models using three statistical metrics: MAPE, CVRMSE, and NMAE. These metrics are widely used in the field because they provide clear and interpretable measures of predictive accuracy in percentage terms, making the results accessible to both technical and non-technical stakeholders.

We selected the harmonic mean (HM) as a representative metric of the overall accuracy of the DT-EL models. This decision was based on the properties of the harmonic mean that make it particularly suitable for averaging ratios and rates. The harmonic mean tends to mitigate the influence of large outliers that can disproportionately affect the arithmetic mean, providing a more balanced and robust measure of performance. This property is crucial when combining error metrics such as MAPE, CVRMSE, and NMAE, which can vary significantly in magnitude.

By using the harmonic mean, we ensure that our evaluation favors models that perform consistently well across all three metrics, rather than models that may excel in one metric but perform poorly in others. This approach supports a comprehensive and balanced interpretation of the results, ultimately helping to identify the most reliable and accurate models for short-term load forecasting.

We hope this additional information provides further insight into our methodology and the rationale behind our choice of the harmonic mean. Thank you again for your valuable feedback.
