# Supplementary material for: Advancing ensemble learning techniques for residential building electricity consumption forecasting: Insight from explainable artificial intelligence
Source: PLoS One. 2024 Nov 14;19(11):e0307654. doi: 10.1371/journal.pone.0307654 (PMC11563398; doi:10.1371/journal.pone.0307654)
Supplement: Attachment — Submitted filename: Response to Review Comments_plos one_Reviwer 2.docx [file pone.0307654.s002.docx]

**Responses to Reviewer 2’s Comments**

We would like to thank Reviewer 2 once again for their constructive feedback during the second round of review. The detailed comments and suggestions provided have been invaluable in enhancing the quality of our manuscript. We have addressed the concerns raised by Reviewer 2 in a careful and thorough manner, striving to incorporate all recommended changes. Please note that the sections of the manuscript that have been newly added or significantly altered are marked in red for clarity. Please find below a detailed response to Reviewer 2's comments and an outline of the corresponding revisions made to the manuscript.

# Reviewer 2

**Comment 1:**

As per Figure 1, the model explanation using SHAP is presented in the form of a summary plot, an interaction plot, a dependence plot, and a heatmap plot. However, in the manuscript, I was unable to find the heatmap plots explaining the SHAP results.

**Response to comment:**

We would like to express our gratitude for your meticulous attention to detail. During the revision period, we made the decision to exclude the heatmap plots from the SHAP results, which resulted in an oversight in the figures presented. We have now corrected this error in Figure 1 to accurately reflect the models used in our analysis. We appreciate this opportunity to clarify and emphasise that SHAP provides superior insights into feature importance compared to other methods such as PDPs and LIME. We would like to thank you for your assistance in enhancing the precision and quality of our manuscript.

**Comment 2:**

To improve the depth of your literature review, I recommend incorporating insights from recent publications (e.g., https://doi.org/10.1016/j.eswa.2024.123729; https://doi.org/10.1109/TSG.2022.3140212).

**Response to comment:**

We are grateful for the recommendation to expand our literature review with insights from recent publications, specifically those mentioned in your comment. We have incorporated discussions on the potential of XAI to expand industrial applications in the energy sector, as highlighted in these studies, into our Discussion section. This addition not only enhances the quality of our manuscript but also identifies potential avenues for future research expansion in our concluding remarks. We would like to thank you for guiding us to enhance the scope and depth of our work, and for setting out a clear pathway for forthcoming investigations.

**Comment 3:**

Tables 5–10: Mention the units of CVRMSE and NMAE.

**Response to comment:**

We appreciate the opportunity to clarify and enhance the readability of our manuscript as suggested. Please be advised that the units for CVRMSE and NMAE are percentages (%) in Tables 5–10. Additionally, we have extended this clarification to Tables 11 to 14, where the units are also in percentages (%). Thank you for your helpful comments that have guided these improvements.
